# A Case of Exuberant Type 2 Pneumocyte Hyperplasia in a Young Man with Recurrent Spontaneous Pneumothorax: A Diagnostic Pitfall

**DOI:** 10.70352/scrj.cr.26-0177

**Published:** 2026-07-11

**Authors:** Fumihiko Hirai, Seiya Kato, Taichi Nagano, Yoshiaki Fujimoto, Kosuke Hirose, Huanlin Wang, Jun Okadome, Takuya Honboh, Noboru Harada, Hiroyuki Ito, Takuo Hayashi, Noriaki Sadanaga

**Affiliations:** 1 Department of Surgery, Saiseikai Fukuoka General Hospital, Fukuoka, Fukuoka, Japan; 2 Division of Pathology, Saiseikai Fukuoka General Hospital, Fukuoka, Fukuoka, Japan; 3 Department of Human Pathology, Juntendo University Graduate School of Medicine, Tokyo, Japan

**Keywords:** exuberant type 2 pneumocyte hyperplasia, spontaneous pneumothorax, differential diagnosis, adenocarcinoma

## Abstract

**INTRODUCTION:**

Exuberant type 2 pneumocyte hyperplasia is a rare histopathological pattern of reactive epithelial change associated with spontaneous pneumothorax, in which preoperative clinical findings and radiological images usually show only nonspecific bullae and blebs. However, it is difficult to differentiate this entity from other conditions, especially pulmonary epithelial tumors, creating significant diagnostic pitfalls in postoperative histopathological evaluation.

**CASE PRESENTATION:**

A 28-year-old man presented with recurrent left-sided spontaneous pneumothorax. Preoperative chest CT revealed subpleural blebs at the left lung apex and in the left lower lobe, without pulmonary nodules or mass lesions. Video-assisted thoracoscopic bullectomy was performed to remove 2 lesions. On gross pathological examination, bullae were observed just below the pleura, consistent with the cause of pneumothorax. In addition, histological examination revealed multiple nodular lesions several millimeters in size within the pulmonary parenchyma, and the airspaces were collapsed due to proliferation of bronchioloalveolar epithelium and accumulation of alveolar macrophages. Despite apparent cytological atypia and high proliferative activity demonstrated by immunohistochemistry (Molecular Immunology Borstel-1 index: 60%), the distinctive histopathological features supported the diagnosis of exuberant type 2 pneumocyte hyperplasia rather than primary or metastatic neoplasia. The patient has been well for over a year after surgery without recurrence.

**CONCLUSIONS:**

Spontaneous pneumothorax is a common condition, but it can also occur secondary to tumors. It is important to differentiate exuberant type 2 pneumocyte hyperplasia from lung tumors, especially atypical adenomatous hyperplasia and adenocarcinoma. Careful histological examination of resected surgical specimens is essential even in cases suspected of primary spontaneous pneumothorax without radiologically apparent tumor-like lesions, to avoid misdiagnosis and overtreatment.

## Abbreviations

AAH
: atypical adenomatous hyperplasia
AE1/AE3
: acidic epithelial keratin (type I)/basic epithelial keratin (type II)
CD1a
: cluster of differentiation 1a
CD68
: cluster of differentiation 68
CK7
: cytokeratin 7
DAD
: diffuse alveolar damage
*EGFR*
: epidermal growth factor receptor
EVG staining
: Elastica van Gieson staining
HE staining
: hematoxylin and eosin staining
HMB-45
: human melanoma black-45
LAM
: lymphangiomyomatosis
MIB-1
: molecular immunology Borstel-1
MMPH
: multifocal micronodular pneumocyte hyperplasia
mTOR
: mammalian target of rapamycin
TSC
: tuberous sclerosis complex
TTF-1
: thyroid transcription factor-1
VATS
: video-assisted thoracoscopic surgery

## INTRODUCTION

Primary spontaneous pneumothorax typically affects young, otherwise healthy individuals and is most commonly associated with rupture of subpleural blebs or bullae; it is generally regarded as a benign clinical condition. On the other hand, secondary spontaneous pneumothorax is associated with various underlying lung diseases, including those showing high morbidity and mortality.^1)^ Surgical management is directed mainly toward the prevention of pneumothorax recurrence and diagnostic clarification.

Nevertheless, a wide variety of histopathological changes have been documented in lung and pleural tissues resected for pneumothorax, including emphysema, fibrosis, inflammatory reactions, and reactive epithelial proliferations.^2)^

Type 2 pneumocyte hyperplasia represents a universal regenerative response to alveolar epithelial injury and is frequently observed in conditions such as DAD and organizing pneumonia. While usually mild, this proliferation of bronchioloalveolar cells can occasionally be so marked that it mimics pulmonary neoplasia such as AAH and adenocarcinoma, particularly those with lepidic or solid growth patterns, creating significant diagnostic pitfalls.^3)^

Shilo et al. first described exuberant type 2 pneumocyte hyperplasia and highlighted this diagnostic pitfall in the setting of spontaneous pneumothorax.^4)^ However, there have been few subsequent case reports of this disease entity, and the etiology and pathogenesis are still unclear. Here, we report a case of exuberant type 2 pneumocyte hyperplasia in a young man with recurrent spontaneous pneumothorax, emphasizing the importance of careful clinicopathological correlation.

## CASE PRESENTATION

A 28-year-old man presented with a sudden onset of left-sided pleuritic chest pain and dyspnea. He had a prior history of conservatively treated spontaneous pneumothorax and was a non-smoker without relevant family history. Physical examination revealed mildly decreased breath sounds on the left, and laboratory tests were unremarkable.

**Fig. 1:**
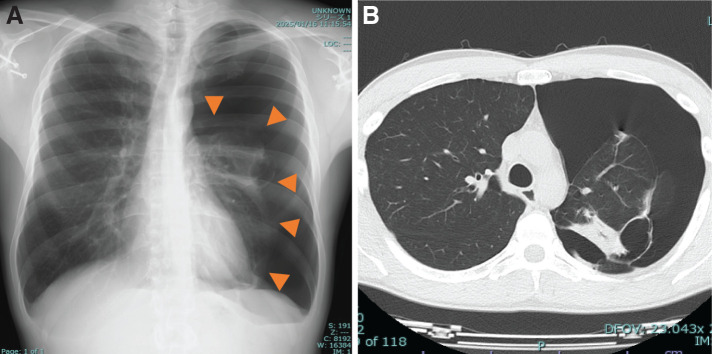
Radiographic findings. (**A**) Chest radiography demonstrated a large left-sided pneumothorax (arrowheads). (**B**) Preoperative chest CT revealed subpleural bullae at the left lung apex as well as in the left lower lobe.

Chest radiography demonstrated a large left-sided pneumothorax without evidence of underlying lung disease (**Fig. 1A**). Preoperative chest CT revealed subpleural bullae at the left lung apex as well as in the left lower lobe. No pulmonary nodules, mass lesions, or areas of consolidation were identified (**Fig. 1B**).

Given the history of multiple recurrences, surgical intervention was elected to prevent further episodes. The patient underwent VATS with wedge resection of the apical and lower lung segments containing the blebs. No abnormal nodules or suspicious tumorous lesions were identified intraoperatively.

**Fig. 2:**
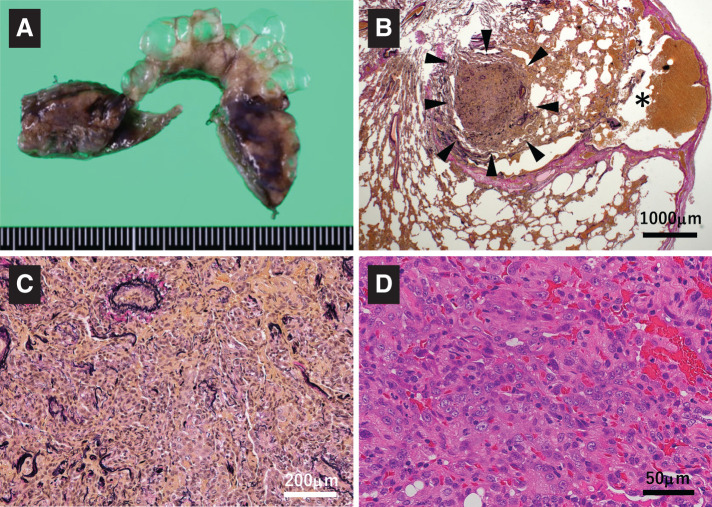
Histopathological overview of the surgical material. (**A**) Macroscopic finding of the partially resected lung tissue showing bulla formation. (**B**) Low-power view of the histology showing the nodular proliferative lesion and emphysematous changes (EVG staining). (**C**) Medium-power view of the histology showing collapsed alveolar spaces and disrupted elastic fibers in the nodular lesion (EVG staining). (**D**) High-power view of the histology showing the nodular lesion composed of proliferation of type 2 pneumocytes and mesenchymal cells (HE staining). Scale bars are indicated in each panel. EVG staining, Elastica van Gieson staining; HE staining, hematoxylin and eosin staining

**Fig. 3:**
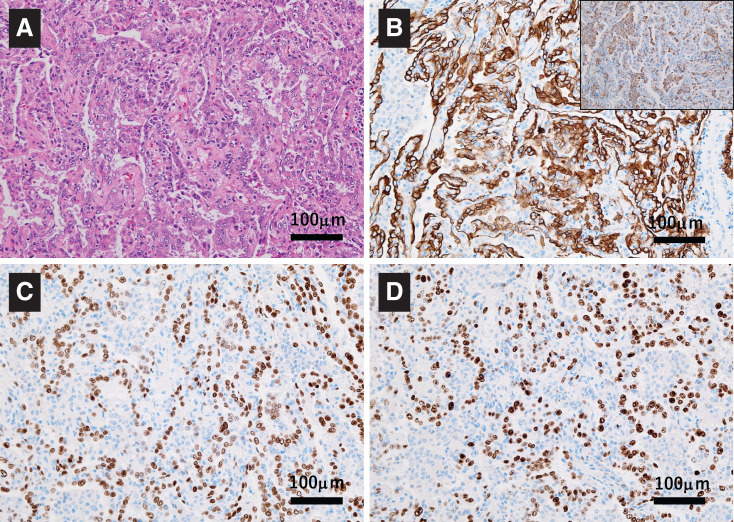
Proliferation of type 2 pneumocytes in the nodular lesion. (**A**) HE staining, medium-power view. Alveolar spaces are lined by type 2 pneumocytes with swollen nuclei. Immunohistochemical stains for AE1/AE3 (**B**), TTF-1 (**C**), and MIB-1 (**D**) highlight type 2 pneumocytes with high proliferative activity. The inset of panel **B** shows CD68-positive macrophages in the alveolar spaces. Scale bars are indicated in each panel. AE1/AE3, acidic epithelial keratin (type I)/basic epithelial keratin (type II); CD68, cluster of differentiation 68; HE staining, hematoxylin and eosin staining; MIB-1, molecular immunology Borstel-1; TTF-1, thyroid transcription factor-1

Histopathologically, gross examination of the resected specimens from both the upper and lower lobes revealed wedges of lung parenchyma with subpleural emphysematous changes and small cystic spaces consistent with blebs and bullae (**Fig. 2A**). Pleural fibrous thickening and nonspecific chronic inflammation with a tissue repair process were observed, consistent with recurrent bullous disease as usually observed.^2)^ In addition, indurated nodules several millimeters in size with unclear borders were observed on the cut surface, and they were microscopically composed of collapsed alveolar spaces with marked proliferation of type 2 pneumocytes (**Fig. 2B–2D**). In some foci, the proliferation formed densely packed epithelial aggregates imparting a solid-like appearance, whereas in other areas the pneumocytes spread along preserved alveolar septa in a lepidic-like growth pattern. Abrupt transitions to normal alveolar lining were observed at the periphery of such lesions. Pneumocytes in the lesions were represented by cuboidal to columnar cell shapes with enlarged nuclei, vesicular chromatin, and occasional prominent nucleoli (**Figs. 2D** and **3A**).

Immunohistochemical staining showed cytoplasmic positivity for AE1/AE3 (pan-cytokeratin, an epithelial cell marker; **Fig. 3B**) and CK7 (a low molecular weight isoform of cytokeratin), and nuclear positivity for TTF-1 (**Fig. 3C**) in the proliferating epithelial cells, confirming their pneumocytic nature. Accumulation of CD68-positive alveolar macrophages was also observed within the air spaces (**Fig. 3B**, inset). No positive staining for HMB-45 or CD1a was noted (data not shown). Regarding epithelial cells, the proliferation index determined by MIB-1 staining was as high as 60% (**Fig. 3D**). However, the cytological features of these epithelial cells lacked overt malignant characteristics such as marked nuclear pleomorphism, hyperchromasia, nuclear stratification, or irregular nuclear contours. A few mitotic figures were noted. No invasive growth pattern, fibrous thickening of the alveolar wall, or desmoplastic stromal reaction was identified. Taken together, we made a diagnosis of exuberant reactive type 2 pneumocyte hyperplasia rather than pulmonary neoplastic lesions such as AAH or adenocarcinoma.

The postoperative course was uneventful, and the patient was discharged in stable condition. Postoperative systemic imaging with CT was performed to exclude occult malignancy or other underlying diseases, and no additional abnormalities were detected. The patient has remained free of recurrence during follow-up for over a year.

## DISCUSSION

This case illustrates a diagnostically challenging but benign reactive process encountered in the setting of recurrent primary spontaneous pneumothorax without any specific predisposing clinical conditions. Shilo et al. reported a series of young male patients with spontaneous pneumothorax in whom prominent pneumocyte hyperplasia closely mimicked pulmonary adenocarcinoma. This entity was termed exuberant type 2 pneumocyte hyperplasia and represents an exaggerated epithelial response to localized alveolar injury and collapse, conditions frequently present in pneumothorax, particularly in recurrent cases.^4)^ Similar to their series, our patient was young, had no radiological evidence of a pulmonary mass, and demonstrated histological changes occurring in a background of lung injury rather than neoplasia. Preservation of alveolar architecture, lack of destructive invasion, and abrupt transitions to normal epithelium strongly favored a reactive interpretation despite dysplastic-appearing epithelial proliferation.

Various forms of acute and chronic pulmonary injury induced by non-neoplastic inflammatory or reactive conditions may promote type 2 pneumocyte hyperplasia.^5,6)^ Among the differential diagnoses, MMPH is particularly important. MMPH is most commonly associated with TSC, often in conjunction with LAM, and is characterized by multiple, well-circumscribed pulmonary nodules composed of bland type 2 pneumocytes.^7,8)^ Functional loss of TSC genes and subsequent activation of the mTOR pathway have been reported to contribute to benign neoplastic proliferation of pneumocytes in MMPH.^9)^ Radiologically, MMPH typically presents as numerous bilateral ground-glass or solid nodules. Histologically, it shows thickened alveolar septa lined by proliferating pneumocytes with abundant alveolar macrophages.^7–9)^ As with exuberant type 2 pneumocyte hyperplasia, differentiation from AAH and lepidic-type lung neoplasia is essential. Although HMB-45 is frequently positive in lesions associated with TSC, including LAM, it is typically negative in MMPH and therefore may not be useful for distinguishing it from exuberant type 2 pneumocyte hyperplasia.^10)^ The Ki-67 labeling indices reported in MMPH are generally low, reflecting its indolent and hamartomatous nature.^11)^ In contrast, the present case showed no history of tuberous sclerosis or involvement of other organs, while the alveolar epithelial proliferation was more pronounced, with mild nuclear atypia and a high proliferation index. A comparison between MMPH and the present case is summarized in **Table 1**. This case highlights the diagnostic difficulty of distinguishing exuberant type 2 pneumocyte hyperplasia from pulmonary epithelial neoplasms in patients undergoing surgery for spontaneous pneumothorax. Type 2 pneumocyte hyperplasia is generally regarded as a reactive process secondary to alveolar injury; however, when the proliferation becomes extensive and exhibits cytologic atypia, it may closely mimic AAH, adenocarcinoma *in situ*, or even invasive adenocarcinoma. In the present case, the lesion demonstrated nodular proliferation of atypical type 2 pneumocytes with an increased MIB-1 labeling index, raising concern for a neoplastic process. Nevertheless, the absence of destructive stromal invasion, significant nuclear pleomorphism, and radiologically detectable nodules favored a reactive rather than malignant lesion.

**Table 1:** Comparison between MMPH and the present case

Feature	MMPH	Present case
Typical age	Young to middle-aged	28 years
Association	TSC	None
Clinical setting	Incidental radiological finding	Recurrent spontaneous pneumothorax
Radiological findings	Multiple bilateral pulmonary nodules	No nodules; bullae and blebs at the left apex and lower lobe
Distribution	Multifocal, bilateral	Localized, subpleural
Background lung injury	Usually absent	Not recognized
Growth pattern	Uniform pneumocytes, septal thickening	Lepidic- and solid-like proliferation
Cytological atypia	Minimal	Difficult to distinguish from tumors
MIB-1 labeling index	Low	Markedly elevated (60%)
Interpretation	Hamartomatous	Reactive

MIB-1, molecular immunology Borstel-1; MMPH, multifocal micronodular pneumocyte hyperplasia; TSC, tuberous sclerosis complex

Previous studies have reported that elevated MIB-1 expression may also be observed in benign or reactive pulmonary conditions, including hypersensitivity pneumonitis, sarcoidosis, and reparative epithelial proliferations associated with lung injury.^12,13)^ These findings suggest that increased proliferative activity alone should not be interpreted as definitive evidence of malignancy. In particular, reactive type 2 pneumocyte hyperplasia occurring adjacent to bullae or areas of alveolar collapse may reflect repeated epithelial injury and regeneration associated with spontaneous pneumothorax. Chronic mechanical stress, localized inflammation, and repeated alveolar damage may contribute to exuberant epithelial proliferation and cytologic atypia.

Differentiation between exuberant type 2 pneumocyte hyperplasia and pulmonary epithelial tumors such as AAH and lepidic adenocarcinoma is problematic. Exuberant type 2 pneumocyte hyperplasia may exhibit high proliferative activity and atypical nuclear morphology, as observed in our case.^3,4)^ The etiology of this entity remains unclear; however, reactive pneumocyte hyperplasia is generally considered to result from lung injury, as observed in DAD, interstitial pneumonia, and other pulmonary diseases.^4)^ Hyperplasia of alveolar epithelial cells with bizarre nuclear morphology is often seen during the repair phase of DAD, and metaplastic epithelium in interstitial pneumonia is frequently difficult to distinguish from neoplastic proliferation.^14)^ Shilo et al. speculated that underlying incidental lung injury in exuberant type 2 pneumocyte hyperplasia may trigger excessive reactive hyperplasia of type 2 alveolar epithelium.^4)^ Therefore, evidence of lung injury in the clinical history or pathological findings may aid in differential diagnosis. Nevertheless, histopathological findings alone are often insufficient to distinguish this lesion from lung tumors, and clinical correlation is essential to confirm its benign nature.^4)^ In the present case, although clear signs of acute lung injury were not evident, the absence of autonomous excessive proliferation or tissue destruction characteristic of malignancy on imaging and postoperative pathological examination supported a non-neoplastic process. Furthermore, tumor-specific genetic alterations, such as *EGFR* mutations, have been reported to be useful markers for distinguishing lung carcinoma from reactive pneumocyte hyperplasia and may assist in differential diagnosis, particularly in middle-aged and elderly patients.^15)^

It remains unclear whether exuberant type 2 pneumocyte hyperplasia represents a cause of spontaneous pneumothorax or a secondary tissue reaction related to rupture and subsequent repair. In recurrent primary spontaneous pneumothorax, activation of matrix metalloproteinases in type 2 pneumocytes has been suggested to contribute to the structural fragility of the lung parenchyma.^16)^ In our case, emphysematous changes and bulla formation were observed adjacent to the nodular proliferative lesions (**Fig. 2B**). The mechanism underlying excessive type 2 pneumocyte proliferation in exuberant type 2 pneumocyte hyperplasia remains unknown. However, it has been hypothesized that this lesion may precede the onset of pneumothorax and function as a check-valve mechanism leading to expansion of air spaces, similar to the mechanism proposed in MMPH.^17)^

Exuberant type 2 pneumocyte hyperplasia has been predominantly reported in younger patients with primary spontaneous pneumothorax, suggesting that it is closely associated with reparative changes following alveolar injury in this population. In the present case, the patient was also relatively young (28 years old), which is consistent with previously reported trends. However, the true age distribution of this entity remains incompletely characterized, as most published cases involve young individuals and reports in middle-aged or elderly patients are extremely limited. This may reflect both a genuine epidemiological tendency and potential under-recognition in older patients.

From a clinical standpoint, recognition of this lesion in older individuals is particularly important. In this age group, the incidence of pulmonary adenocarcinoma and its precursor lesions, such as AAH, is significantly higher, and reactive type II pneumocyte proliferations may closely mimic these entities histologically. Therefore, in elderly patients, exuberant type 2 pneumocyte hyperplasia may present a greater diagnostic challenge and increase the risk of overdiagnosis of malignancy, especially in limited biopsy or intraoperative frozen sections. Careful correlation of histological findings with radiologic and clinical features is essential to avoid misinterpretation and to ensure appropriate management.

## CONCLUSIONS

Exuberant type 2 pneumocyte hyperplasia is a benign tissue lesion characterized by excessive proliferation of the alveolar epithelium that is rarely identified in surgical specimens resected for spontaneous pneumothorax and is generally associated with a favorable prognosis. Even in young patients with apparently primary pneumothorax and no radiologically detectable lesions, careful clinical and histopathological evaluation can prevent misinterpretation of reactive changes as malignancy and thereby avoid unnecessary additional treatment.

## DECLARATIONS

### Availability of data and materials

The datasets supporting the conclusions of this article are included within the article.
